# Prevalence of hearing loss and tinnitus among school-age children: a cross-sectional analysis of the PICTURE cohort study

**DOI:** 10.3389/fpubh.2026.1703400

**Published:** 2026-03-25

**Authors:** Jan Piotrowski, Katarzyna Pazdro-Zastawny, Katarzyna Połtyn-Zaradna, Krzysztof Kujawa, Katarzyna Zatońska, Agnieszka Matera-Witkiewicz, Katarzyna Kiliś-Pstrusińska, Tomasz Zatoński

**Affiliations:** 1 Department of Otolaryngology, Wroclaw Medical University, Wrocław, Poland; 2 Division of Population Studies and Prevention of Noncommunicable Diseases, Faculty of Health Sciences, Wroclaw Medical University, Wrocław, Poland; 3 Statistical Analysis Centre, Wroclaw Medical University, Wrocław, Poland; 4 Screening Biological Activity Assays and Collection of Biological Material Laboratory, Faculty of Pharmacy, Wroclaw Medical University, Wrocław, Poland; 5 Clinical Department of Paediatric Nephrology, Faculty of Medicine, Wroclaw Medical University, Wrocław, Poland

**Keywords:** cohort studies, hearing loss, otoacoustic emissions, prevalence, tinnitus

## Abstract

**Background:**

Hearing loss and tinnitus in children are increasingly recognized as important public health concerns with potential long-term consequences for development and communication. The aim of this study was to determine the prevalence of hearing loss and tinnitus in children, and to examine the concordance between parental subjective assessments and otoacoustic emission (OAE) results. Secondary aims were to investigate the association between hearing loss and sociodemographic factors, parents’ level of education, and to evaluate the effectiveness of different diagnostic methods in detecting pediatric hearing impairment.

**Methods:**

This cross-sectional study involved 1,250 children aged 7–17 years. Participants underwent tympanometric, hearing threshold and OAEs testing and were divided into two groups according to age. Parents completed a questionnaire regarding hearing loss and presence of tinnitus.

**Results:**

The prevalence of positive hearing screening results was 3.5%, with a higher incidence in younger children, (the difference was not statistically significant). The prevalence of hearing loss was 4.5% among boys and 2.5% among girls (*p* = 0.097). A statistically significant discrepancy was observed between parental reports of hearing status and OAE results (*p* = 0.006). The prevalence of tinnitus in children and adolescents aged 7–17 years was 4.7%, with no significant statistical difference observed between age groups. Abnormal results in OAE were not associated with tinnitus (the results were not statistically significant). A statistically significant association was observed between hearing loss detected by OAE and caregiver education (*p* = 0.040, *Z* = −2.05), and between hearing loss and increased healthcare costs (*p* = 0.015). The effectiveness of hearing loss detection differed significantly between the diagnostic methods used (*p* = 0.030), with OAE testing showing the highest detection rate.

**Conclusion:**

This study identifies a measurable prevalence of hearing loss and tinnitus among children, with a greater burden of hearing impairment observed in younger age. There is need for systematic, school-based hearing screening programs and improved parental and educator awareness of early hearing loss indicators. These findings support the integration of standardized pediatric hearing monitoring into public health strategies and underscore the importance of addressing socioeconomic disparities in access to early diagnosis and intervention.

## 1 Introduction

Hearing loss is a common global health condition, affecting approximately 430 million people, including 34 million children (1). Hearing impairment in children can significantly impact speech and language development, education, social functioning, and cognitive outcomes (2). Sensorineural hearing loss (SNHL) arises from both genetic and environmental factors, such as ageing, socioeconomic status, noise exposure, cardiovascular health, and ototoxic medications (3). Hearing loss is also a known risk factor for tinnitus, which appears more frequently in children with hearing impairment than in those with normal hearing (4, 5). While the prevalence of subjective tinnitus in adults is well documented, population-based data on tinnitus and hearing loss in children particularly in Poland remain scarce. Existing estimates suggest that tinnitus affects 8.5–21% of children aged 5–17 years, with severe cases reported in 2.7% (6), but comprehensive, representative data are lacking. Early identification of hearing loss through screening is essential for timely intervention and rehabilitation, minimizing its long-term effects on development and quality of life (7, 8). Poland has a long-standing tradition of auditory screening for newborns using otoacoustic emissions (OAEs) and auditory brainstem response (ABR) testing (9). However, systematic screening programs for school-aged children are limited and vary in implementation across healthcare institutions (7, 10–13). Pure-tone audiometry and OAEs are commonly used internationally and allow detection of both conductive and sensorineural deficits (14, 15). Despite these efforts, there is a clear gap in population-based data on hearing loss and tinnitus among Polish school-aged children. Systematic monitoring is needed to accurately estimate prevalence, evaluate screening effectiveness, and guide public health interventions, including diagnostic and rehabilitation services. Addressing this gap will support evidence-based policies to mitigate the impact of hearing loss and tinnitus in childhood (16). Hearing screening for school-age children in European countries varies depending on the healthcare system and availability of resources, but the most commonly used methods are pure-tone audiometry and otoacoustic emissions (10). In the UK, screening is primarily performed using pure tone audiometry, while in Sweden and Germany, a combination of pure tone audiometry and otoacoustic emissions is used (11–13). In Poland, hearing screening programs for school-aged children including otoacoustic emissions and pure tone audiometry are conducted by some medical and public health institutions, such as the Institute of Physiology and Pathology of Hearing (IFPS) (7). Pure-tone audiometry allows the assessment of auditory sensitivity throughout the auditory pathway and differentiates between conductive and sensorineural deficits (14). OAE testing has been regularly proposed as a potential screening method due to its simplicity, short examination time, impartial results, and minimal requirement for patient cooperation (14, 15).

There is a strong need for systematic monitoring of children’s hearing status, which would facilitate more accurate studies of hearing loss prevalence and determination of screening test sensitivity and specificity. These steps will, in turn, enhance our ability to study the effects of screening and treatment interventions, supporting the development of guidelines on the screening, diagnostic, and rehabilitation services needed to mitigate the impact of childhood hearing loss (16).

This study aims to estimate the prevalence of hearing loss and tinnitus in school-aged children, and to assess the influence of sociodemographic factors such as the average monthly expenditure on private medical visits for the entire household and the parents’ level of education on the occurrence of hearing loss.

## 2 Study design and methods

The data showed in this study was acquired from the pro-health Population Cohort Study of Wroclaw Citizens - PICTURE program, a cohort study involving school-aged children and their caregivers, which was started in 2019 and was formed and arranged by the Wroclaw Medical University. Data from children, adolescents, and their caregivers were gathered between 2019 and 2023. The PICTURE study is one of the most comprehensive longitudinal cohort studies conducted in a population of children and their parents in Poland. The primary objective of the study is to analyze and assess the health status and lifestyle of children and their parents. The secondary aim is to identify and analyze environmental and social factors that may influence lifestyle and the occurrence of risk factors for non-communicable diseases. Wroclaw Medical University specialists created special questionnaire which was used in this survey study.

The PICTURE study used a two-stage recruitment process. In the first stage (2019), 3,750 children aged 7–14 years were randomly selected from the PESEL database, stratified by age and sex, and registered as residents of Wrocław, Poland (inclusion criteria). In the second stage (2021), an additional 1,250 children were randomly selected using the same procedure, resulting in a total sample of 5,000 invited participants. Invitations were sent by mail. Individuals who did not respond to the invitation were excluded from the study. Ultimately, 1,250 children and adolescents aged 7–17 years participated in the study. Data were collected from children, adolescents, and their 1,250 caregivers between 2019 and 2023. The project was designed to conduct longitudinal analysis, forming a Wroclaw-based cohort. Data was collected in a standardized manner, at two-year intervals, with annual follow-up via telephone contact. The recruitment process of the Polish cohort at baseline, along with the detailed methodology, was described by Zatońska et al. (17). This analysis represents the cross-sectional component of the PICTURE study. This study focusses on pediatric population and provides an assessment of hearing loss and tinnitus in school-age children in Wrocław, Poland. Each child underwent a full physical otolaryngological examination. The study group was divided into two age groups: younger children (7–12 years) and older children (13–17 years).

Participants underwent tympanometric, hearing threshold and otoacoustic emission testing. Tympanometry was performed using a 226 Hz probe tone, with ear canal pressure varied from −150 to +150 daPa to provide quantitative information about the presence of fluid in the middle ear, mobility of the middle ear system, and ear canal volume. In our study, three types of tympanometric curves were described: Type A, Type B, and Type C, to assess middle ear function in the examined children. An audiometric evaluation took place in a soundproof audiometric chamber to determine if the patient’s hearing levels fall within normal limits and was always performed by the same professional staff. The participants were fitted with headphones into which audiometer-generated tones of varying frequency and intensity were played, first on the ear that the subject identified as the better-hearing ear. The values obtained were automatically recorded as audiograms, separately for the right and left ear. Hearing loss was expressed in the audiogram as decibel hearing level (dB HL), and threshold curves of mean values in dB HL were generated for each frequency for the specified categories. Audiologic testing was performed by a technician who had no knowledge of each child’s hearing status. The collected data included air conduction thresholds in each ear at frequencies of 0.5 kHz, 1 kHz, 2 kHz, 4 kHz and 8 kHz. The pure-tone average for bone conduction in each ear was calculated for each patient as the average of bone conduction thresholds at 0.5 kHz, 1 kHz, 2 kHz, and 4 kHz. The airbone gap in the affected ear was calculated as the difference between air conduction and bone conduction thresholds at 0.5 kHz, 1 kHz, 2 kHz, and 4 kHz. Otoacoustic emission (OAE), a physiological test that measures the cochlea’s (outer hair cell) response to a stimulus is an effective screening tool for detecting inner and middle ear abnormalities, as it assesses the mechanical performance of the hearing receptor in order to detect cochlear hearing loss. Cochlear function was assessed using distortion product otoacoustic emissions (DPOAE) measured across frequencies from 1 to 6 kHz. Each parent of a child participating in the study was asked to complete a questionnaire that included questions regarding possible hearing difficulties, including observed signs of hearing loss. The responses were compared with the results of an objective hearing assessment conducted using OAE, allowing for the evaluation of the consistency between parental observations and the actual auditory function of the child. As part of the comparative analysis, audiometric test results were compared with tympanometry findings, and tympanometry results were further compared with otoacoustic emissions outcomes, in order to assess the consistency between the different diagnostic methods.

Hearing loss was defined as the average intensity of the softest sound that can be heard in one or both ears at frequencies between 500 and 4,000 Hz being greater than or equal to 20 dB. Furthermore, hearing loss was categorized into six categories based on average intensity, including mild hearing loss (thresholds at 20–34 dB), moderate hearing loss (thresholds at 35–49 dB), moderately severe hearing loss (thresholds at 50–64 dB), severe hearing loss (thresholds at 65–79 dB), very severe hearing loss (thresholds at 80–94 dB), and total hearing loss (thresholds over 95 dB) (18).

Tinnitus according to literature was defined as the conscious awareness of noise or tone for which there is no corresponding external acoustic source, and individuals are diagnosed with a tinnitus disorder if this awareness results in distress (19). Literature suggests that tinnitus may be associated with neurodevelopmental changes in the auditory system, highlighting the intersection between these conditions (19). Subjective tinnitus being the most prevalent form of tinnitus and is thought to arise from underlying neuronal dysfunction. As it cannot be directly observed or objectively measured by clinicians, its assessment relies primarily on patient self-report. Consequently, there is currently no objective method for reliably identifying the presence of subjective tinnitus or for quantifying the level of distress it causes (20). Caregiver education level was divided into: primary, vocational, secondary, higher education.

The study was anonymous and voluntary. The Local Ethics Committee of Wrocław Medical University (KB-667/2019) approved the study. All children and adolescents and their caregiver took part in the project voluntarily and could discontinue their involvement at any time without consequences. The exact character and aim of the project were explained to all participants; in each case, consent to take part in the study was formally expressed. The ethical standards established in the 1964 Declaration of Helsinki and its later adjustments were a priority during conducting this study.

## 3 Statistical analysis

Statistical analysis was performed using the Statistica v.13 software package (StatSoft Inc., Tulsa, OK., USA). Depending on the nature of the data, the following statistical tests were applied: Cochran-Armitage test (for trend in the proportion of a binary variable) using the R package”Desk Tools”(0.99.58), McNemar’s test (for differences in proportions between two dependent variables), Pearson’s Chi-squared dependence test with Yates’ correction (for differences in proportions between two independent variables)., the Wilcoxon rank sum test, and the normal distribution test (Lilliefors test if *n* > 50, Shapiro–Wilk test if *n* ≤ 50). For all tests, *p* < 0.05 was considered statistically significant.

## 4 Results

### 4.1 The prevalence of hearing loss detected by otoacoustic emissions (OAEs) according to age and sex

OAE data were available for 1,148 children. Hearing loss, defined as abnormal OAE results, was detected in 40 children, corresponding to a prevalence of 3.5% (40/1,148), while normal hearing was observed in 96.5% (1,108/1,148) of participants (Table 1). When stratified by age group, abnormal OAE results were identified in 4.1% of younger children (n = 28/690) and in 2.6% of older children (12/458) (Table 2). The effect of age groups on abnormal OAE result prevalescence was not statistically significant (*p* = 0.256), with an odds ratio of 1.56 (95% CI: 0.80–3.23). The prevalence of hearing loss was slightly higher in males 4.5% (26/584) compared to females 2.5% (14/564), (*p* = 0.097) (Table 3).

**Table 1:** Hearing loss based on otoacoustic emissions (OAE).

Category	Total count (*N*)	Percentage (%)
Abnormal (hearing loss)	40	3.5
Normal hearing	1,108	96.5

**Table 2:** The frequency of abnormal otoacoustic emission (OAE) results was compared across age groups.

Age group	Hearing loss (Abnormal)	Normal hearing (Normal)	Hearing loss (%)	Normal hearing (%)
Younger	28	662	4.1	95.9
Older	12	446	2.6	97.4

**Table 3:** Prevalence of hearing loss by gender.

Hearing status	Female (*N*)	Male (*N*)	Female (%)	Male (%)
Abnormal	14	26	2.5	4.5
Normal	550	558	97.5	95.5

### 4.2 Comparison between parental questionnaire responses and otoacoustic emissions

Parental questionnaire data and OAEs results were available for 1,134 children. There was a statistically significant difference (McNemar test *p* = 0.006) indicating a discrepancy between parents’ subjective assessments of their children’s hearing and the objective OAE test results. Among children with abnormal OAE results, hearing loss was reported by parents in 2.6% (1/38) of cases, whereas 97.4% (37/38) were reported as having normal hearing. Among children with normal OAE results, parental reports indicated hearing loss in 1.5% (16/1,096) of cases (Table 4).

**Table 4:** Comparison between parental questionnaire responses and otoacoustic emissions (OAE) McNemar’s test, *p* < 0.05.

OAE result	Reported hearing loss	Normal hearing	Reported hearing loss (%)	Normal hearing (%)
Abnormal	1	37	2.6	97.4
Normal	16	1,080	1.5	98.5

### 4.3 Tinnitus prevalence was assessed in relation to age and gender, with variations observed across both factors

Tinnitus data were available for 1,214 children. Tinnitus was reported by 4.7% of children (57/1,214), while 95.3% (1,157/1,214) did not report this symptom. Tinnitus was reported by 29 children in the younger group 4.0% (29/725) and 5.7% children in the older group (28/489) This difference was statistically insignificant (*p* = 0.209). However, odds ratio (OR) was 1.46 (95% CI: 0.85–2.49), indicating a tendency for older children to have higher odd of tinnitus (Table 5). The prevalence of tinnitus by sex showed that 4.8% of girls (29/602) and 4.6% of boys (28/612) reported tinnitus, which difference was statistically insignificant (*p* = 0.949). The estimated odds ratio (OR) was 0.95 (95% CI: 0.55–1.62), suggesting no significant effects of sex on tinnitus prevalence (Table 6).

**Table 5:** Prevalence of tinnitus by age group.

Age group	Tinnitus		Total	Prevalence (%)
	No	Yes		
Younger	696	29	725	4.0
Older	461	28	489	5.7
Total	1,157	57	1,214	4.7

**Table 6:** Prevalence of tinnitus by gender.

Sex	Tinnitus		Total	Prevalence of tinnitus (%)
	No	Yes		
Female	573	29	602	4.8%
Male	584	28	612	4.6%
Total	1,157	57	1,214	4.7%

### 4.4 Tinnitus and hearing loss assessed by otoacoustic emissions

Among the children with available data (1,214), tinnitus was reported by 4.7% participants (57/1,214), whereas 95.3% children (1,157/1,214) did not report tinnitus. When tinnitus occurrence was analyzed in relation to hearing status assessed by otoacoustic emissions, 7.9% (3/38) children with abnormal OAE results reported tinnitus, compared with 4.8% children (53/1,096) with normal OAE results. The association between tinnitus and hearing loss detected by OAE was not statistically significant (*p* = 0.635).

### 4.5 Hearing loss and caregiver education level, and average monthly amount of money spent on private medical visits for the whole household

Data on caregiver education level and OAE results were available for 1,126 children. The proportion of abnormal OAE results varied across education levels, ranging from 0.0% (0/3) among caregivers with primary education to 9.7% (3/31) among those with vocational education, 4.6% (11/241) with secondary education, and 2.8% (24/852) with higher education (Table 7). The Cochran–Armitage test demonstrated a statistically significant trend between caregiver education level and decrease in abnormal OAE result frequency (*p* = 0.040).

**Table 7:** Comparison between caregiver education level and Otoacoustic Emissions (OAE) Cochran-Armitage test *p* < 0.05.

Caregiver education level	Number of children with abnormal results	Number of children with normal results	Proportion of abnormal results (%)	Proportion of normal results (%)
Primary	0	3	0.0	100.0
Vocational	3	28	9.7	90.3
Secondary	11	230	4.6	95.4
Higher	24	828	2.8	97.2

**Figure 1:**
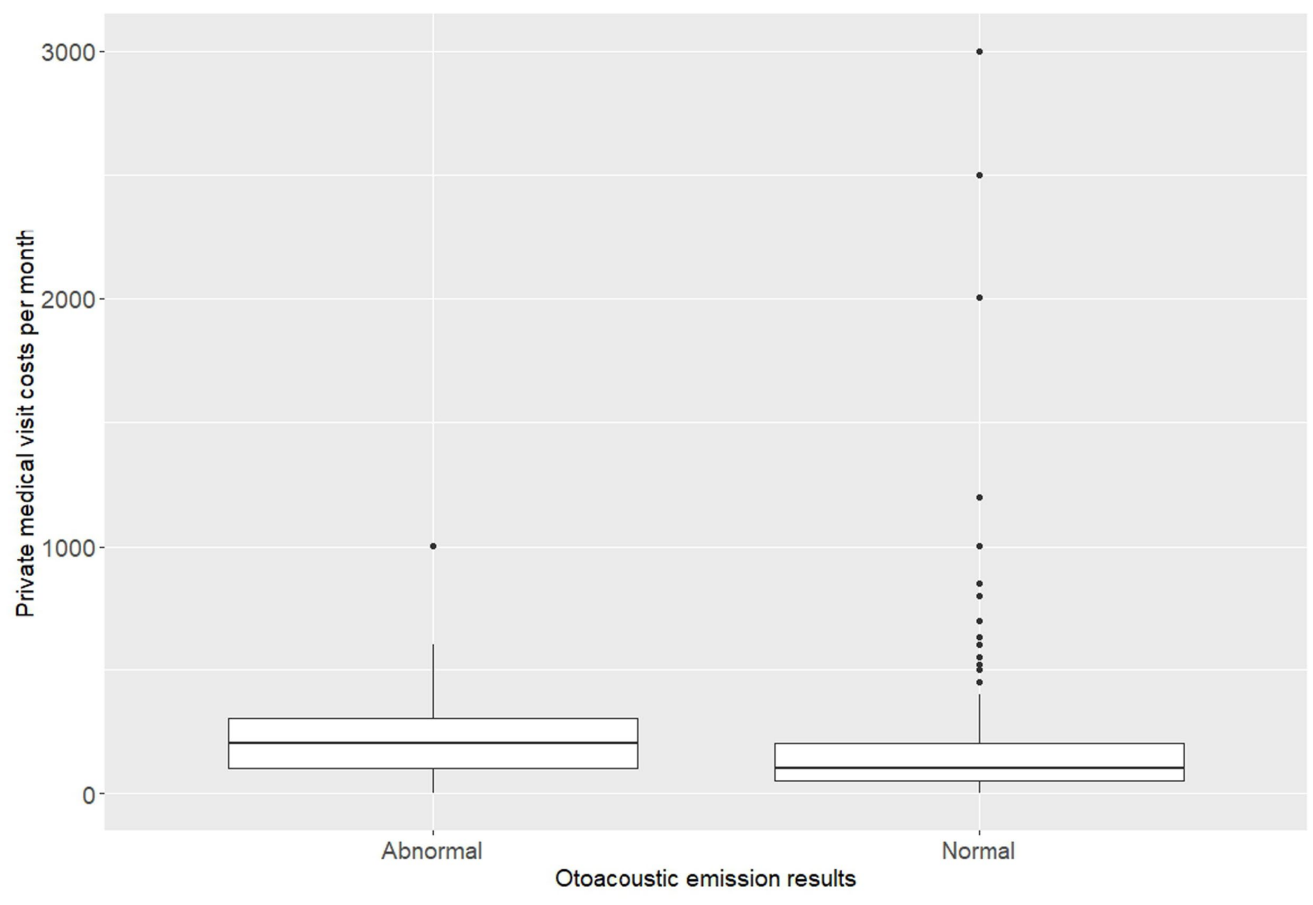
The private medical visit costs per month in children with normal and children with abnormal otoacoustic emission.

Analysis of the average monthly amount of money spent on private medical visits for the entire household revealed a significant difference between children with abnormal and normal OAEs results. The normality test indicated a non-normal distribution of such healthcare costs in both groups (Shap < 0.001 in normal OAEs group, and *p* = 0.008 in abnormal OAEs group); therefore, a non-parametric test was used for the comparisons between these groups. The Wilcoxon rank sum test revealed that children with hearing loss had significantly higher expenses compared to those with normal results, with *p* = 0.015 and as illustrated in Figure 1, the values of the median, as well as the lower (Q1) and upper (Q3) quartiles, were higher in children with hearing loss than in those without (Figure 1).

### 4.6 Comparison of acoustic tympanometry, otoemission and tonal audiometry

Pure-tone audiometry data were available for 1,202 children, OAE data for 1,148 children, and tympanometry data for 1,205 children. Hearing loss was detected in 2.7% (32/1,202) using pure-tone audiometry, 3.5% (40/1,148) using OAEs, and 1.7% (21/1,205) using tympanometry (Table 8). Detection rates differed significantly across the three diagnostic methods, with the highest rate observed for OAE (3.5%) compared with tympanometry (1.7%) (*p* = 0.030).

**Table 8:** Comparison of hearing loss detection rates across three audiological methods: pure-tone audiometry (PTA), otoacoustic emissions (OAE), and tympanometry.

Method	Hearing loss (N)	Normal hearing (N)	Hearing loss (%)	Normal hearing (%)
Pure-tone audiometry (PTA)	32	1,170	2.7	97.3
Otoacoustic emission (OAE)	40	1,108	3.5	96.5
Tympanometry	21	1,184	1.7	98.3

## 5 Discussion

Our study revealed, that hearing impairment detected using OAEs was observed in 3.5% of children and was more prevalent in boys than in girls. A higher prevalence of hearing loss was identified among younger children compared to older children (4.1% vs. 2.6%), which is consistent with findings reported by Feder et al. (21), who observed a comparable prevalence of 3.4% and similarly noted a higher burden in younger age groups.

The increased prevalence of hearing disorders observed in younger children might be due to several factors (22). Younger children have a higher susceptibility to ear infections compared to older age groups, primarily due to anatomical and immunological immaturity. Recurrent upper respiratory tract infections, which may lead to Eustachian tube dysfunction, impaired middle ear ventilation, otitis media and transient conductive hearing loss (23–25).

In our study, the prevalence of hearing loss was 2.7% as determined by pure-tone audiometry. The prevalence of hearing loss in our study was higher than that reported in Chinese research (0.17%) (26). However, Skarżyński et al. (27) reported a 9.4% prevalence of hearing loss in a population-based cross-sectional study involving 67,416 children aged 6–13 years. Such discrepancies across studies are likely attributable to differences in sample size, screening protocols, and definitions of hearing loss.

In our study, we observed a statistically significant difference, indicating a discrepancy between parents’ subjective assessments of their children’s hearing and the objective OAE results. This finding suggests that caregiver reports alone may not reliably identify hearing impairment. Only a few studies have tested parents’ awareness of their children’s hearing loss in OAEs. Pure-tone audiometry tests were more commonly used for comparison. In a study by Lo et al. (28), parents of children with otitis media with effusion showed a low ability to predict hearing loss in a pure-tone audiometry, correctly identifying only 12% of cases, despite the high prevalence of the condition and its associated risks. These findings emphasize the need for objective audiological screening and enhanced parental education regarding early signs of hearing impairment.

The prevalence of tinnitus in our study was assessed at 4.7%, with no significant differences observed between boys and girls. Kim et al. (29) in a a cross-sectional study of 940 students aged 10–12 years in Seoul, Korea, revealed that 41 (4.4%) experienced continuous tinnitus. Raj-Koziak et al. (30) in a population-based epidemiological study of 43,064 school-age children from the general pediatric population in Warsaw, Poland demonstrated that 3.1% of children were affected by tinnitus. Another study by Nemholt et al. (31) investigated tinnitus prevalence in Danish children aged 10 to 16 years. The cross-sectional study included 501 participants and found a tinnitus prevalence of 66.9% (31).

In our study, younger children reported tinnitus less frequently than older children. A similar trend was found in the study of Meijers et al. (32) and Kim JS (33). Using different index questions to identify people with tinnitus, applying variations in sample size, or assessing differences in the level of suffering will have a significant impact on the outcome in research (5, 30, 32).

The etiology of tinnitus in children is multifactorial and not limited to peripheral auditory dysfunction (34). In the present study, tinnitus was reported without a significant association with hearing loss detected by otoacoustic emissions. Previous research indicates that many children are aware of tinnitus-related sensations and may be particularly sensitive to internal auditory perceptions, especially in the presence of psychosocial stressors. Kim et al. (29) demonstrated a significant association between tinnitus in children and increased levels of stress and trait anxiety, highlighting the role of psychological factors in the development and persistence of tinnitus symptoms. These findings support the consideration of psychosocial factors when evaluating tinnitus in children and underscore the importance of a holistic approach that includes both audiological assessment and evaluation of emotional wellbeing. In addition to psychosocial influences, emerging evidence suggests that lifestyle and dietary factors may contribute to the development of tinnitus in young people. For example, Tomanic et al. (35) reported a significant association between dietary habits and tinnitus among 15–19-year-old school-aged adolescents, demonstrating that the risk of tinnitus increased with higher consumption of white bread, carbonated beverages, and fast food. Conversely, a strong negative correlation was observed between tinnitus occurrence and the consumption of fresh fruit and vegetables, suggesting that a ‘healthy’ diet, either alone or alongside other factors, may have a protective effect.

Although no significant association between tinnitus and hearing loss was found in our study, considering both conditions together may provide insights into potential neurodevelopmental and psychosocial interactions, as suggested by existing literature (29, 30, 35).

Our research indicated that a low level of parental education appeared to be a risk factor for children with a hearing impairment. Previous research has shown that a lower level of parental education is a risk factor for hearing loss (26). Parental education level may be related to informed decisions before or during pregnancy and child raising. People with less education are more likely to be exposed to second-hand smoke, pollution and noise (34). Such unhealthy lifestyle factors may result in poorer hearing outcomes for their future offspring (34, 36, 37). We also found that the average monthly amount of money spent on private medical visits for the whole household was higher in children with hearing loss. There may be a few main reasons for the increased monthly expenditure on private medical visits for children with hearing loss. Firstly, early diagnosis of hearing loss results in more visits needed for effective treatment and rehabilitation. Secondly, late diagnosis of hearing loss results in higher costs associated with advanced hearing damage, which may require more costly interventions such as cochlear implants or hearing aids (38). Thirdly, long queues in public healthcare (National Health Service) force parents to use more expensive private appointments (39). Further research is needed to examine this problem more closely.

We compared the effectiveness of three hearing screening methods: pure-tone audiometry, tympanometry and OAE. Our study revealed that OAE is the most effective method for detecting hearing loss, Trzaskowski et al. (40) similarly suggest that o OAEs can be efficiently used to test the hearing of schoolchildren; however, PTA and tympanometry tests should be used to supplement this approach. A study by Nunes et al. (41) found that of the three procedures evaluated, OAE had the best suitability (sensitivity, specificity and predictive values) for use in a school setting. Moreover, it has a shorter duration of use and greater acceptability among students and can detect the most common causes of hearing impairment in the school context, although it has limitations in distinguishing between conductive and sensorineural problems. On the other hand, a study by Śliwa et al. (42) showed that the best performance was observed in automated four-tone audiometry, followed by tympanometry and OAE procedures. Combining audiometry and tympanometry procedures improved screening performance. Further research is needed to determine the most effective method for assessing children’s hearing. This research should incorporate standard stimulus levels, response criteria and definitions of hearing loss (43). The combined audiometry, OAE, and tympanometry protocol provides the best balance between detecting hearing loss and being practical for use in schools (41, 42, 44).

## 6 Conclusion

Our findings highlight the importance of implementing hearing screening programs for the early detection of hearing impairment. Screening should be conducted not only during the neonatal and infant periods but also incorporated as a routine component of school-based medical care. The results underscore the need for systematic monitoring of hearing status in children, as well as for increased awareness among parents, educators, and healthcare providers regarding the significance of hearing loss.

Limited concordance between parental subjective assessments and objective hearing screening outcomes emphasizes the necessity of objective audiological testing rather than reliance on caregiver reports alone. Moreover, the identification of tinnitus in a substantial proportion of children supports the inclusion of routine tinnitus-related questions in pediatric health check-ups. Health education initiatives accompanying hearing screening programs may further improve awareness, promote hearing-protective behaviors, and facilitate timely access to diagnostic and rehabilitative services.

Finally, the observed associations between hearing loss, caregiver education level, and increased private healthcare expenditures highlight the role of sociodemographic factors in pediatric hearing health and access to care. Further population-based studies conducted across diverse regional and socioeconomic contexts are warranted to enable meaningful comparisons of prevalence estimates and to inform evidence-based public health strategies for the prevention and early detection of hearing loss and tinnitus in children.

### 6.1 Study limitations

There are few limitations of this cross-sectional study that should be considered. First, the study included both unilateral and bilateral hearing lossunder the general category of hearing impairment, which may have limited the specificity of the analysis. Another limitation was the lack of detailed categorization of pure-tone average thresholds, including these measures could have provided a more comprehensive characterization of the type and degree of hearing loss. The survey relied on binary (yes/no) parental responses to the questions “Does the child have a hearing loss?” and “Does the child have tinnitus?,” which does not allow parents to express uncertainty or gradations of symptoms. This could be a potential source of bias, and a broader set of response options would have been preferable.

Data on tinnitus were based solely on subjective self-reports from questionnaires, without confirmation by objective diagnostic methods. As this study was based on a screening test, hearing loss and tinnitus were identified through preliminary assessments, and the group of children with these conditions represents cases that require further comprehensive diagnostic evaluation. In addition, the variable amount of data available for individual participants may have affected the statistical power of certain comparisons and could have introduced inaccuracies.

## Data Availability

The original contributions presented in the study are included in the article/supplementary material, further inquiries can be directed to the corresponding author.
